# Exosomal miR-22-3p targets p53 to regulate endometrial epithelial cell function in the pathogenesis of endometriosis

**DOI:** 10.1007/s10735-026-10966-1

**Published:** 2026-09-04

**Authors:** Congxiang Yu, Yuefei Li, Gele Qi, Qiao Qiao

**Affiliations:** 1 Department of Obstetrics and Gynecology, Affiliated Hospital of Inner Mongolia Medical University, Hohhot, 010050 Inner Mongolia China; 2 Department of Obstetrics and Gynecology, Inner Mongolia Maternal and Child Health Care Hospital, Hohhot, 010020 Inner Mongolia China; 3 Inner Mongolia Medical University, Hohhot, 010050 Inner Mongolia China

**Keywords:** Exosomes, Endometriosis, Endometrial epithelial cells, MiR-22-3p, P53, Apoptosis

## Abstract

Endometriosis (EMs) is a widespread gynecological disease, affecting approximately 6–10% of females of child-bearing age. Despite its significant influence on women’s health, the specific pathophysiology remains unclear. The enrichment of miR-22-3p in exosomes has been implicated in cellular proliferation and migration. This study aimed to elucidate the mechanisms by which exosomal miR-22-3p affects endometrial epithelial cell function in the etiology of endometriosis. The patient samples and clinical data were acquired from the Affiliate Hospital of Inner Mongolia Medical University. Quantitative reverse transcription‒polymerase chain reaction (qRT‒PCR), a miRNA microarray and Western blotting were used to confirm that the level of miR-22-3p was specifically elevated in peripheral blood exosomes from EMs patients. The influence of miR-22-3p on EMs-derived exosomes was determined by Transwell and cell counting kit-8 (CCK-8) assays. Luciferase reporter assays, RNA pull-down assays, and fluorescence in situ hybridization (FISH) were performed to determine whether exosomal miR-22-3p binds to p53. miR-22-3p expression was significantly elevated in peripheral blood exosomes (*p* < 0.001) from patients with endometriosis. ROC curve analysis demonstrated the high sensitivity (93.55%) and specificity (84.85%) of this correlation, suggesting that miR-22-3p is a promising diagnostic biomarker. Endometrial epithelial cell proliferation, migration, and invasion are regulated by exosomal miR-22-3p, which targets and inhibits p53, a crucial tumor suppressor related to cell cycle regulation and apoptosis. Exosomal miR-22-3p regulates endometrial epithelial cell proliferation, migration, and invasion by targeting and inhibiting p53. Combining standard endometriosis treatments with exosome-mediated miR-22-3p targeting of p53 could restore p53 function, induce apoptosis, and reduce disease recurrence, providing a promising therapeutic strategy for endometriosis management.

## Introduction

Endometriosis (EMs) is a prevalent gynecological disease that affects the physical and emotional health of women. The incidence of EMs is increasing, with a worldwide prevalence rate of up to 15% among females of child-bearing age (Moga et al. 2019). Chronic pelvic pain, dyspareunia, dysmenorrhea, and infertility occur in 30% to 50% of EMs patients (Bulletti et al. 2010). EMs negatively affects the quality of life of patients and increases the healthcare burden on society because of increased healthcare and other expenses. EMs patients may present with a wide range of lesions, diverse morphologies, highly invasive metastases, and additional malignant characteristics. In current clinical practice, hormonal medications or surgery are typically used to improve patient symptoms (Prescott et al. 2016). Despite the development of novel medications and improvements in laparoscopic procedures, endometriosis remains incurable. However, the main challenge is the high recurrence rate following conservative surgery.

Consequently, to successfully prevent disease progression and the incidence of adverse effects such as severe side effects, early diagnosis and treatment are critical. Pelvic endometriosis is classified into Stages I–IV (mild–severe), and the illness is often detected 6–9 years after it first manifests. Despite being the “gold standard” for diagnosing endometriosis, patients do not generally accept laparoscopy because of its high cost and invasive nature (Yu et al. 2018). Several investigations have been conducted worldwide with the aim of achieving early diagnosis and therapy. Nevertheless, no specific biological markers have been identified, and the pathophysiology of EMs remains unclear. The main goals of EMs research include understanding the pathophysiology of endometriosis and identifying molecular markers for precise type-specific diagnosis.

Exosomes are small extracellular vesicles that transmit signals between cells and are largely stable in fluids, including serum, saliva, and urine (Mishra et al.^2021^, Mao and Anastasi 2010). Exosomes encapsulate chemicals that impact the pathophysiology of target cells, namely, microRNAs and signaling proteins (Kalluri and LeBleu 2020). Exosomes extracted from epithelial cells contribute to several pathophysiological processes, including antigen presentation, immune responses, replicative cellular senescence, and cell migration and differentiation (Sha et al. 2019, Huang et al. 2022, Jiang et al. 2022). Exosomes have also been shown to control immunoinflammation, stimulate tumor angiogenesis, alter the premetastatic tumor microenvironment, and activate fibroblasts in the induced tumor microenvironment. Research has shown a substantial correlation between exosomes and EMs growth.

Exosomes were effectively extracted from the ascites of patients with endometriosis in 2020 by Hannah et al. (Zondervan et al. 2018). These authors reported a positive association between the number of exosomes and disease severity. Proteome analysis revealed that the number of exosomes was greater in the endometriosis group than in the healthy group. In 2021, Mishra (Bedaiwy et al. 2017) demonstrated that endometrial-associated exosomes are involved in the emergence of endometriosis and other endometrium-related dysfunctions by controlling inflammation, angiogenesis, and the local microenvironment. A variety of circulating exosomal miRNAs exhibit specificity for endometriosis tissues, and exosomes are thought to contain miRNAs, which could be biomarkers for numerous diseases (Colombo et al. 2014). Wang et al. reported that the expression of miRNA-188-5p and miR-NA-4741 derived from plasma exosomes was lower in patients with endometriosis and that these biological markers may be useful for diagnosis (Chen 2021). However, the functions of exosome-derived miRNA-188-5p and miR-NA-4741 in the evolution of endometriosis remain unclear (Chen et al. 2019). Previous studies have shown that exosomes can contribute to endometriosis through a range of different mechanisms. Nevertheless, little is known about how exosomes control the invasion, translocation, and proliferation of endometrial epithelial cells. However, the role of exosomes on endometrial epithelial cell migration has not been studied. No definitive molecular marker for the type-specific diagnosis of endometriosis has been identified.

Here, miR-22-3p was specifically selected for investigation based on the following rationale. First, our preliminary miRNA microarray analysis of peripheral blood exosomes from endometriosis patients and healthy controls identified miR-22-3p as one of the most significantly upregulated miRNAs. Second, bioinformatic analysis using TargetScan and miRDB databases predicted p53, a critical regulator of cell cycle and apoptosis, as a potential target of miR-22-3p. Third, recent studies have implicated miR-22-3p in the regulation of stromal cell functions in endometriosis, but its specific role in endometrial epithelial cells—the primary cell type responsible for lesion formation—remains unexplored. These combined lines of evidence led us to hypothesize that exosomal miR-22-3p may regulate endometrial epithelial cell function through p53 targeting in endometriosis pathogenesis. In the present study, exosomal miR-22-3p was detected in the serum of endometriosis patients, and in vitro models were used to determine the molecular mechanisms through which exosomal miR-22-3p affects endometrial epithelial cell function in the etiology of endometriosis.

## Materials and methods

The human samples and related clinical data were obtained from the Affiliate Hospital of Inner Mongolia Medical University. This study was supported by the Science and Technology Program of the Joint Fund of Scientific Research for the Public Hospitals of Inner Mongolia Academy of Medical Sciences (20242024GLLH0281, Hohhot China), the Youth Fund Program of the Natural Science Foundation of the Inner Mongolia Autonomous Region (2022QN0826, Hohhot China), the Program for Health Science and Technology in the Inner Mongolia Autonomous Region (202202202, Hohhot China), and the Youth Cultivation Project of Inner Mongolia Medical University (YKD2021QN014). The Ethics Committee of the Affiliated Hospital of Inner Mongolia Medical University approved this study (Approval No. FS(KY2023092).

### Inclusion and exclusion criteria

Patients with suspected endometriosis scheduled for elective laparoscopy were enrolled. Inclusion criteria for the EMs group were: (1) age 24–45 years; (2) regular menstrual cycles (25–35 days); (3) laparoscopically confirmed endometriosis, staged according to the revised American Fertility Society (r-AFS) classification (stages I–IV); (4) no hormonal therapy for at least 3 months prior to surgery; and (5) written informed consent.

Exclusion criteria for the EMs group were: (1) concomitant gynecological malignancies; (2) pelvic inflammatory disease; (3) uterine fibroids requiring intervention; (4) ovarian cysts of non-endometriotic origin; (5) pregnancy or lactation; (6) recent use (within 3 months) of hormonal contraceptives, GnRH agonists, or other hormonal therapies; (7) autoimmune diseases; and (8) acute or chronic infections.

For the healthy control group, inclusion criteria were: (1) age-matched women (24–45 years) undergoing routine gynecological examination; (2) regular menstrual cycles; (3) no history of endometriosis or other gynecological disorders; (4) no hormonal therapy for at least 3 months; and (5) written informed consent. Exclusion criteria for controls were: (1) presence of pelvic masses or endometriotic lesions on transvaginal ultrasound; (2) chronic pelvic pain; (3) any gynecological surgery within the past year; (4) hormonal therapy within 3 months; (5) pregnancy or lactation; and (6) any chronic inflammatory or autoimmune condition.

### Patient consent and sample acquisition

Peripheral blood samples were obtained from women aged 24–45 years who had undergone elective laparoscopy for suspected endometriosis after providing informed consent. Blood samples were centrifuged for 20 min at 4 °C and 3000×g. The supernatant was carefully extracted and stored at − 80 °C. The experimental group included patients with confirmed endometriosis (*n* = 30), whereas the control group included age-matched healthy women without endometriosis or hormone-related disorders (*n* = 30). Prior to recruitment, all the patients had not received hormone therapy for at least three months, and all the patients had regular menstrual cycles.

### Cell culture

*Cell recovery* Immortalization endometrial epithelial cells (purchased from Fenghui Biotechnology, Hunan, China) were thawed in a 37 °C water bath. Afterward, 3 mL of prewarmed culture medium (CORNING, USA) was added, the sample was mixed well with a pipette and transferred to a 15 mL centrifuge tube. After being centrifuged for 5 min at 800 rpm, the supernatant was aspirated, and the cell pellet was resuspended in fresh medium. Endometrial epithelial cells were seeded in 10 cm culture plates and incubated in an incubator under standard conditions.

*Cell passaging* When the endometrial epithelial cells reached 80–90% confluence, the cells were washed twice with PBS, treated with 2 mL of 2.5% trypsin-EDTA digestion solution, and neutralized by the addition of 8 mL of 10% FBS in RPMI 1640 medium. The cells were subsequently transferred to a 15 mL centrifuge tube. After being centrifuged for 5 min at 800 rpm, the supernatant was aspirated, and the cell pellets were resuspended and counted. Cells were seeded in 10 cm culture plates at a density of 3–5 × 10^5^/mL and incubated under standard conditions.

*Exosomes isolation and identification* Exosomes were extracted from the blood serum of endometriosis patients and healthy controls. Exosome extraction reagent (0.5 volumes) was added, and the mixture was vortexed we and incubated overnight at 4 °C. Ultracentrifugation was performed at 10,000 × g for 1 h at 2–8 °C, and the pellet was resuspended in PBS. The exosomal protein concentration was measured with a Bradford protein assay kit (Wuhan Dr. Tak Bio-Tech Co.). The morphology of the exosomes was examined by transmission electron microscopy (TEM) (SOPTOP OD630K, Shanghai Sunyu Hengping Scientific Instrument Co.). The size range and concentration were analyzed by nanoparticle tracking analysis (NTA) with a Flow NanoAnalyzer (USA). Western blotting was performed to assess the expression of the exosomal markers CD9, CD61, and HSP70 (Abcam, England).

*TEM examination* For TEM examination, exosome pellets were resuspended in 50 µL PBS and fixed with 2.5% glutaraldehyde in 0.1 M phosphate buffer (pH 7.4) for 30 min at room temperature. A 10 µL drop of the fixed exosome suspension was placed on a formvar-carbon-coated copper grid and allowed to adsorb for 2 min. The grid was negatively stained with 2% uranyl acetate for 1 min, air-dried, and examined under an HT7700 transmission electron microscope (HITACHI, Tokyo, Japan) at an accelerating voltage of 100 kV. Images were captured at 100,000× magnification.

*NTA* For NTA, exosome pellets were resuspended in PBS and diluted to an appropriate concentration within the instrument’s optimal detection range (approximately 10^6^–10^8^ particles/mL). The particle concentration was then determined using a Flow NanoAnalyzer (USA), which provides direct measurement rather than preset concentration. Particle size distribution profiles (mean and mode) were recorded for each sample.

*miRNA transfection* Endometrial epithelial cells were seeded in 60 mm culture plates at a density of 1 × 10^6^ cells/well. Twenty-four hours later, cells were transfected with miR-22-3p inhibitor (50 nM) or inhibitor NC (50 nM) using Lipofectamine^®^ 3000 Transfection Reagent (Invitrogen, USA) according to the manufacturer’s protocol. Transfection complexes were prepared in Opti-MEM™ reduced-serum medium (Gibco, USA). After 4 h of transfection, complete culture medium was added. After cells were cocultured with exosomes (20 µg/mL) for 24 h, they were collected for further experiments.

*Cell proliferation assay* A CCK-8 assay kit (Wuhan Doctor Bioengineering Co., China) was used to assess cell proliferation in the control, exosome+inhibitor NC, and exosome+inhibitor groups. Endometrial epithelial cells were seeded in 96-well plates, and proliferation was determined at 0 and 24 h using an enzyme immunoassay microplate reader (Beijing Purang New Technology Co.) by measuring the absorbance at 450 nm.

*Transwell migration assay* For the Transwell migration assay, 24-well Transwell inserts (8 μm pore size; Orange Scientific, Belgium) were coated with 50 µg/mL fibronectin overnight at 4 °C and air-dried. Endometrial epithelial cells were serum-starved for 12 h, then detached, counted, and resuspended in serum-free medium at a density of 1 × 10^5^ cells/mL. Cell suspension (200 µL) was added to the upper chamber. Complete medium (600 µL) supplemented with 20% FBS (Gibco, USA) was added to the lower chamber as a chemoattractant. After 24 h of incubation at 37 °C in 5% CO₂, non-migrated cells on the upper surface of the membrane were gently removed with a cotton swab. Migrated cells on the lower surface were fixed with methanol-acetic acid (3:1) for 15 min and stained with 0.1% crystal violet for 20 min. Stained cells were counted in five randomly selected fields per well under an inverted microscope (SOPTOP OD630K, China) at 200× magnification.

*Reverse transcription–quantitative PCR (RT-qPCR)* According to the manufacturer’s instructions, total RNA was extracted from exosomes and cells with TRIzol reagent (Aidlab, China). qRT‒PCR was completed by using 2× SYBR Green qPCR Master Mix (None ROX; Servicebio). The following primers were used: miR-22-3p, forward-5′-AAGCTGCCAGTTGAAG-3′ and reverse-5′-TGGT TGGTCGTGGAGTCG-3′; and U6: forward-5′-CTCGCTTCGGCAGCACA-3′ and reverse-5′-AACGCTTCACGAATTTGCGT-3′. Relative RNA expression levels were calculated by the 2^–△△Ct^ method and normalized to the expression of U6.

*Western blotting* Proteins were extracted from endometrial epithelial cells, separated and transferred to PVDF membranes (Millipore, USA). The membranes were then blocked with 5% skim milk and incubated with antibodies against CD61 (1:1000; Abcam, England), CD9 (1:1000; 1:1000; Abcam, England), and HSP70 (1:1000; 1:1000; Abcam, England) at 4 °C overnight. Additionally, Western Lightning™ Chemiluminescence Reagent (NEL10300EA) (PerkinElmer, USA), RIPA tissue/cell lysate (Cat# R002) (Beijing Soleberg Technology Co., China) and Western protein marker V II (G2087) (Servicebio) were used. The membranes were subsequently incubated with a horseradish peroxidase-conjugated secondary antibody (Abcam, England) for one hour. ECL (Millipore, USA) solution was subsequently applied to observe the bands. Relative protein expression was quantified by a BioImaging System.

*Dual luciferase reporter gene assay for targeting validation* HEK-293T cells were transfected with P53-WT or P53-MUT constructs and miR-22-3p mimic or mimic-NC. A dual-luciferase reporter system (Promega, Madison, WI, USA) was used to measure luciferase activity 24 hours post-transfection. The predicted miR-22-3p binding site within the p53 3’-UTR corresponds to nucleotides 178–184 (sequence: 5’-…AAGCTGCCAGTTGAAG…-3’). For the mutant construct (p53-UTR-MUT), the seed sequence was mutated to 5’-…TTCGACGGTCAACTTC…-3’ (mutations underlined) to disrupt miR-22-3p binding.

*RNA Pull-down assay* Endometrial epithelial cells were treated with biotin-labeled constructs (Bio-miR-22-3p-wt, Bio-miR-22-3p-mut, or Bio-NC). Streptavidin beads (Biolabs, New England) were used to perform the RNA pull-down assay. P53 levels were detected by RT‒qPCR.

*Fluorescence in situ hybridization (FISH)* FISH probes were synthesized by Saiweier (China), and the experiments were conducted using a Ribo Fluorescent In Situ Hybridization Kit (Boster, USA). Endometriotic cells were fixed, permeabilized, and hybridized overnight with a gossypol-labeled oligonucleotide probe at 37 °C. The nuclei were counterstained with DAPI. Images were subsequently obtained with an Olympus microscope (Japan).

*Statistical analysis* GraphPad Prism (version 6.0) and SPSS software (version 20.0; IBM Corp., Armonk, NY, United States) were used for data analysis. One-way analysis of variance and the Student–Newman–Keuls test were used to compare multiple groups. Two-tailed Student’s t tests were used to analyze the differences between two groups. All experiments were performed in triplicate, and the data are reported as the mean±standard deviation. ***p ≤ 0.001, **p ≤ 0.01, *p ≤ 0.05, and ^ns^ *p* ≥ 0.05. Sample size (*n* = 30 per group) was determined based on preliminary experiments (miR-22-3p expression difference: mean 2.5-fold, SD 1.2, α = 0.05, power = 0.80) using G*Power software (version 3.1). Post-hoc analysis confirmed that this sample size provided > 80% power to detect the observed differences.

## Results

### miR-22-3p expression is higher in exosomes from individuals with endometriosis than in exosomes from the healthy individuals

**Fig. 1:**
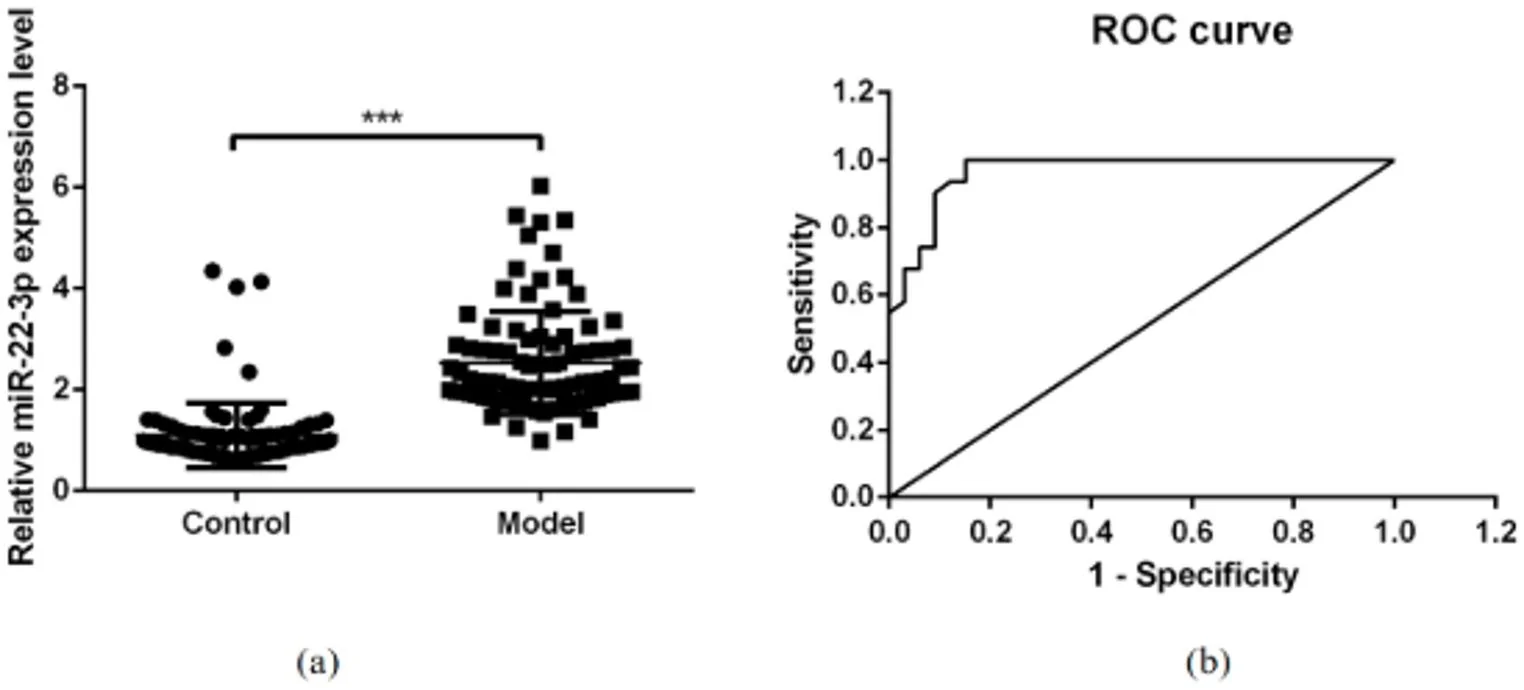
Compared with that in healthy individuals, the expression of miR-22-3p in exosomes in individuals with endometriosis was greater (***p < 0.001) (**a**). The AUC from the ROC curve analysis was 0.9648 (95% CI: 0.921–0.998), with a sensitivity of 93.55% and specificity of 84.85% at the optimal cutoff value (ΔCt = 4.28), indicating high predictive diagnostic value (**b**)

The expression of miRNA-22-3p RNA in exosomes obtained from peripheral blood differed between individuals with and without endometriosis. Compared with healthy individuals (*n* = 30), patients with endometriosis (*n* = 30) had noticeably higher expression of miR-22-3p in peripheral blood exosomes (***p < 0.001, Fig. 1a). The AUC from the ROC curve analysis was 0.9648 (95% CI: 0.921–0.998), with a sensitivity of 93.55% and specificity of 84.85% at the optimal cutoff value (ΔCt = 4.28), indicating high predictive diagnostic value(Fig. 1b).

### Identification of exosomes

**Fig. 2:**
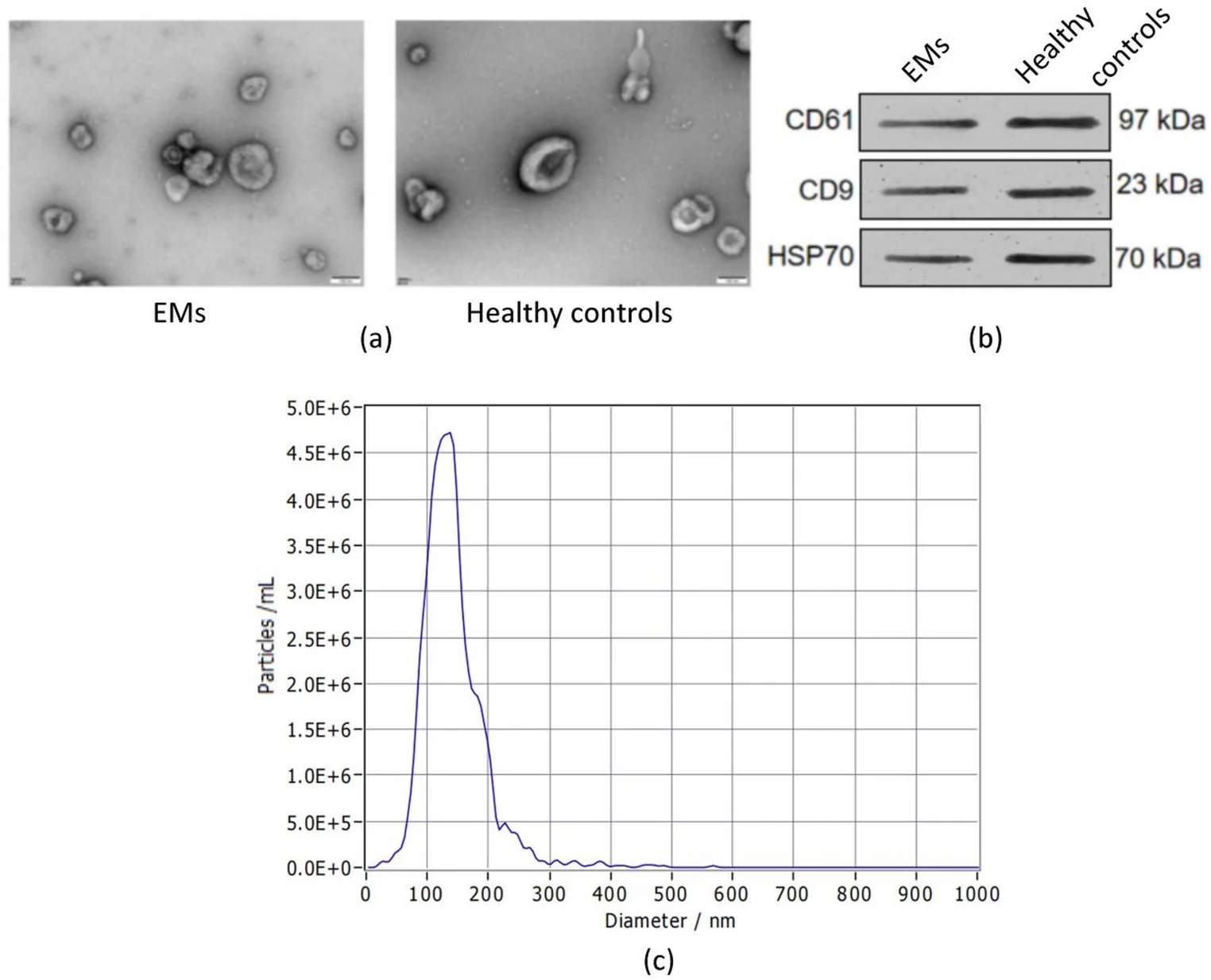
Exosomes were extracted from the peripheral blood of individuals with endometriosis and healthy controls (**a**). The results of western blotting revealed that the expression of CD 61, CD9, and HSP70 was significantly greater in the EMs group than in the healthy control group (**b**). The results of NTA detecting the particle size distribution (**c**)

Exosomes were extracted from the peripheral blood of individuals with and without endometriosis. As shown in Fig. 2a, TEM analysis revealed that the morphology of the exosomes from both endometriosis patients and healthy women was elliptical. The western blot results demonstrate that exosome extraction from both the experimental and control groups was successful. The expression of CD 61, CD9, and HSP70 was significantly greater in the EMs group than in the healthy control group (Fig. 2b). Furthermore, the exosome content and particle size distribution were investigated using NTA, and both types of exosomes were 150 nm in size. The results verified the effective extraction of exosomes from peripheral blood (Fig. 2c).

### Exosomes regulate the biological functions of endometrial epithelial cells through the insertion of miR-22-3p

**Fig. 3:**
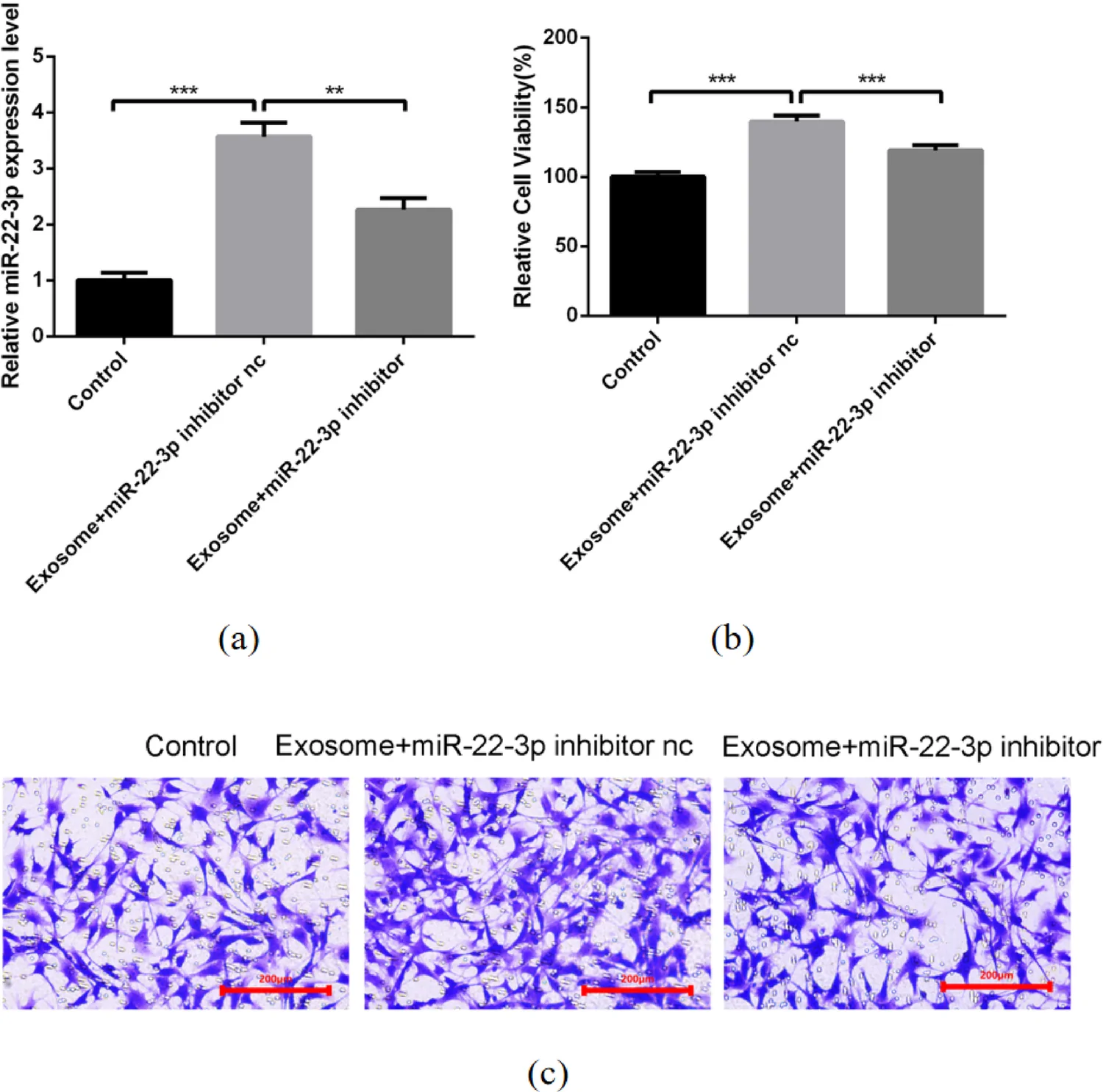
Effects of exosomal miR-22-3p on endometrial epithelial cell proliferation, migration, and invasion. **a** relative miR-22-3p expression in endometrial epithelial cells after treatment with EMs-derived exosomes (Exo) + miR-22-3p inhibitor nc or Exo + miR-22-3p inhibitor (Inhibitor). Cells treated with exosomes + inhibitor nc showed significantly higher miR-22-3p expression than control cells (***p < 0.001). Cells treated with exosomes + miR-22-3p inhibitor showed significantly reduced miR-22-3p expression compared to Exo+inhibitor nc cells (**p < 0.01), confirming successful knockdown. **b** CCK-8 proliferation assay showing that cells treated with Exo+inhibitor NC exhibited significantly increased proliferation compared to control (***p < 0.001), while Exo+inhibitor treatment significantly attenuated this effect (**p < 0.001). **c** transwell migration and invasion assays showing that Exo+inhibitor nc treatment significantly increased cell migration and invasion compared to control (***p < 0.001), while Exo+inhibitor treatment significantly reduced these activities (**p < 0.01). All experiments were performed in triplicate. Data are presented as mean ± SD. **p < 0.01, ***p < 0.001 versus control or as indicated. ns, not significant

After endometrial epithelial cells were transfected with the miR-22-3p inhibitor, exosomes were introduced. The cells were split into control, exosome + miR-22-3p inhibitor NC, and exosome + miR-22-3p inhibitor groups. Following RNA extraction from the three cell groups, qPCR was used to measure the expression of miR-22-3p. The expression of miR-22-3p was higher in the exosome + miR-22-3p inhibitor NC group than in the control group (***p < 0.001); however, the expression of miR-22-3p was lower in the exosome + miR-22-3p inhibitor group than in the exosome + miR-22-3p inhibitor NC group (**p < 0.01) Fig. 3a.

As shown in Fig. 3b, the MTT assay revealed that cell proliferation was greater in the exosome+inhibitor NC group than in the control group (***p < 0.001). However, cell proliferation decreased in the exosome+inhibitor group compared with that in the exosome+inhibitor NC group (**p < 0.001).

The Transwell and invasion assays demonstrated greater activity in the exosome+inhibitor NC group than in the control group (***p < 0.001). However, compared with the exosome+inhibitor NC group, cellular activity in the Transwell and invasion assays decreased in the exosome+inhibitor group (**p < 0.01) Fig. 3c.

### miR-22-3p regulated the biological ability of endometrial epithelial cells by targeting p53

**Fig. 4:**
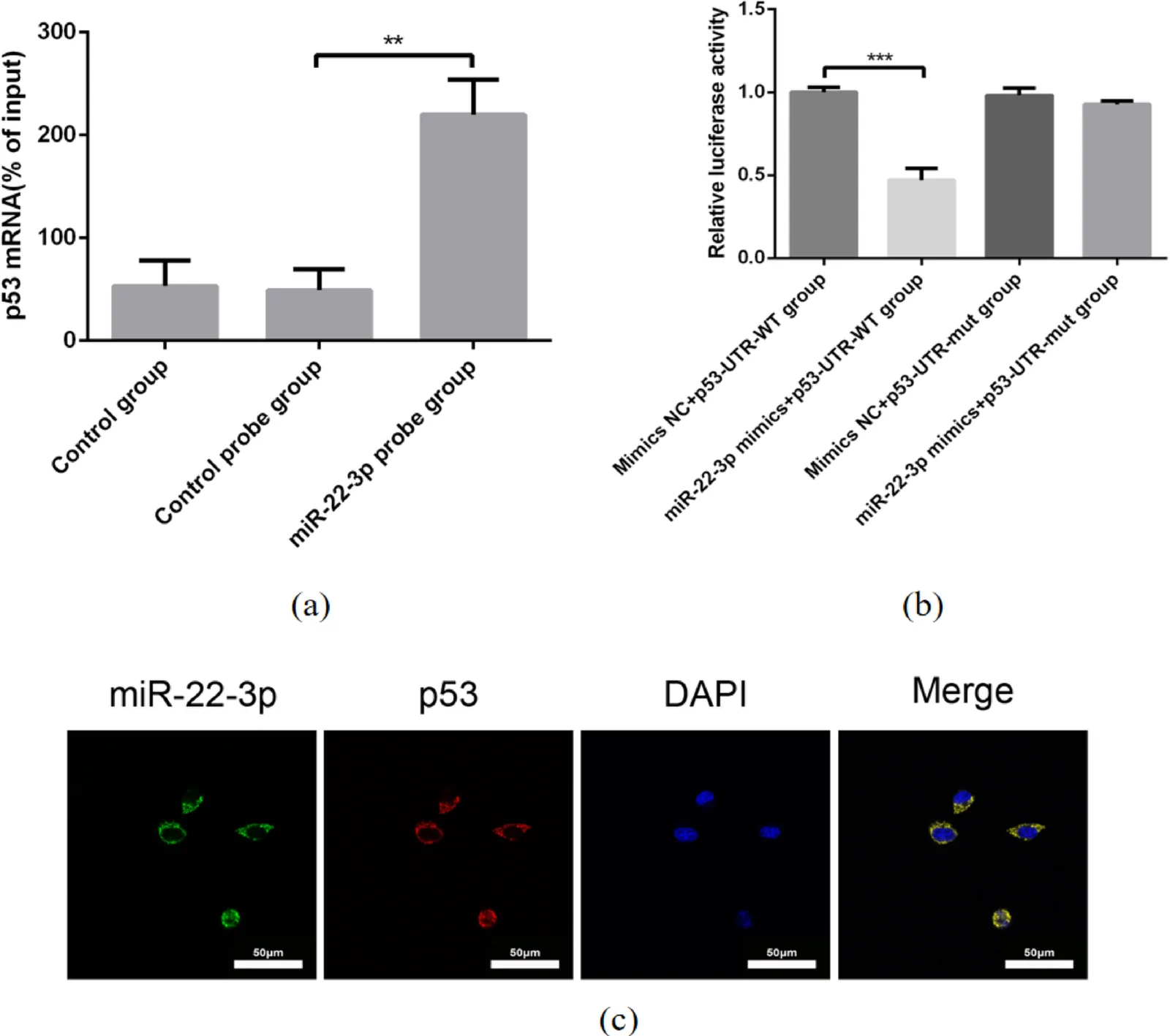
**a** RNA pull-down revealed that miR-22-3p can interact with p53. **b** compared with the NC+p53-UTR-WT group, the miR-22-3p mimic+p53-UTR-WT group had lower relative luciferase activity (*P* < 0.05). However, there were no significant differences between the mimic NC+p53-UTR-WT group and the mimic NC+p53-UTR-mut group or between the miR-22-3p mimic+p53-UTR-mut group (*p* > 0.05), suggesting that miR-22-3p can target and interact with p53 in endometrial epithelial cells. **c** The FISH results indicated that miR-22-3p and p53 colocalized in the cytoplasm

Compared with the NC+p53-UTR-WT group, the miR-22-3p mimic+p53-UTR-WT group had lower relative luciferase activity. However, there were no significant differences between the mimic NC+p53-UTR-WT group and the mimic NC+p53-UTR-mut group or between the miR-22-3p mimic+p53-UTR-mut group (*p* > 0.05) (Fig. 4a), suggesting that miR-22-3p can target and interact with p53 in endometrial epithelial cells. As shown in Fig. 4b, the FISH results indicated that miR-22-3p and p53 colocalized in the cytoplasm (Fig. 4c).

### miR-22-3p targets p53

**Fig. 5:**
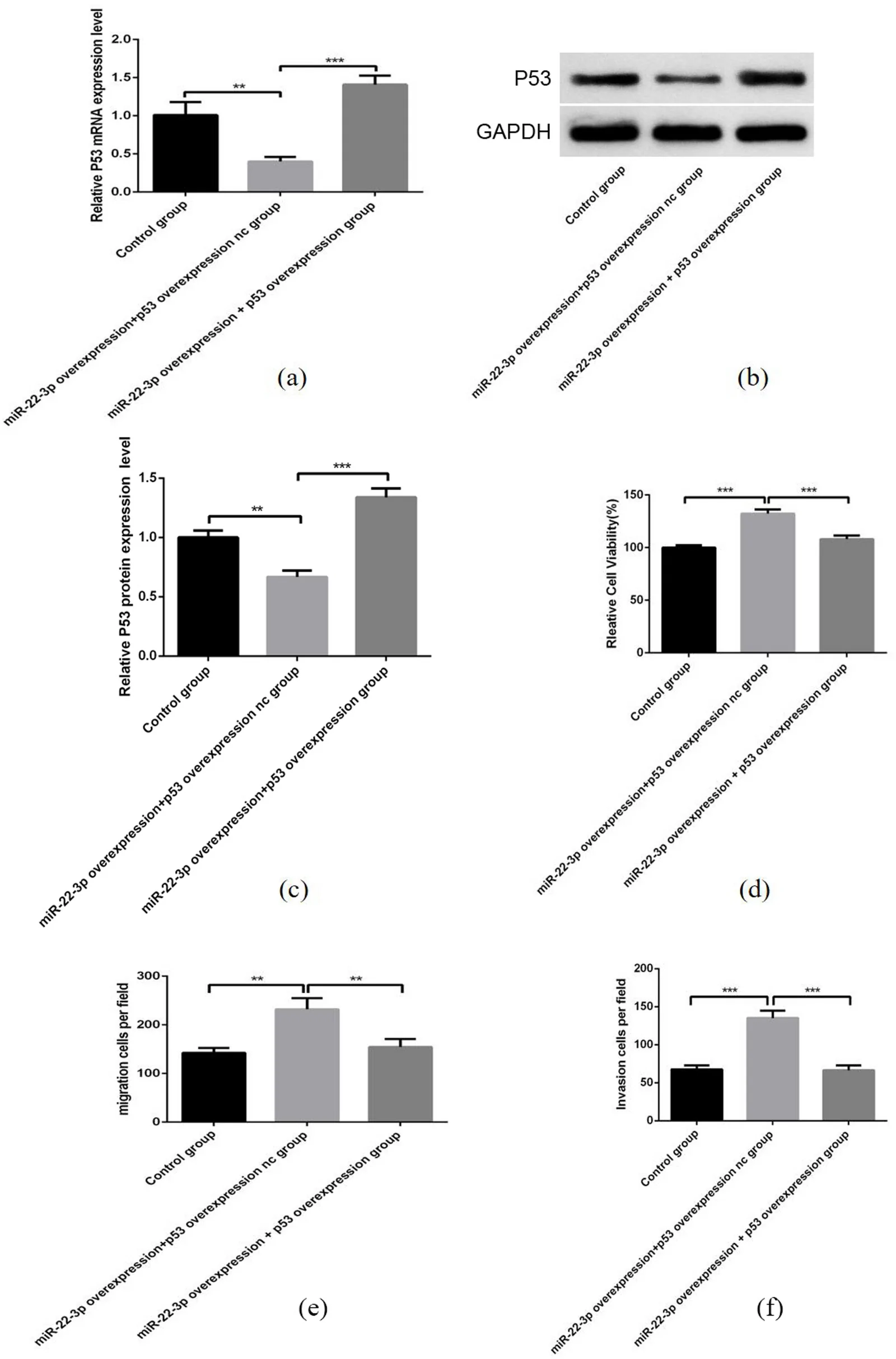
**a** Compared with the control group, P53 expression was lower in the miR-22-3p overexpression+p53 overexpression nc group (*p* < 0.05) but greater in the miR-22-3p overexpression + p53 overexpression group (*p* < 0.01). **b**,** c**,** d** the results of the CCK-8 and Transwell assays demonstrated that compared with the control group, the proliferation, migration and invasive capabilities were greater in the miR-22-3p overexpression + p53 overexpression nc group (*p* < 0.01) but lower in the miR-22-3p overexpression+p53 overexpression group (*p* < 0.01)

Among the many potential mRNA targets of miR-22-3p, p53 was selected for further investigation based on the following considerations. First, p53 is a well-established tumor suppressor that regulates cell cycle arrest, apoptosis, and DNA repair, processes that are critically involved in endometriosis pathogenesis. In addition, reduced apoptosis in ectopic endometrial tissues has been well documented in endometriosis, suggesting that p53 dysregulation may contribute to disease progression. he RT‒PCR results revealed that P53 expression was lower in the miR-22-3p overexpression+p53 overexpression nc group than in the control group (*p* < 0.05). However, compared with that in the miR-22-3p overexpression+p53 overexpression nc group, P53 expression was much greater in the miR-22-3p overexpression + p53 overexpression group (*p* < 0.01, Fig. 5a). The results of the CCK-8 and Transwell assays demonstrated that compared with that in the control group, proliferation, migration, and invasion were greater in the miR-22-3p overexpression + p53 overexpression nc group (*p* < 0.01, Fig. 5b, c). Conversely, compared with that in the control group, the miR-22-3p overexpression + p53 overexpression group presented reduced proliferation, migration, and invasion capacity (*p* < 0.01, Fig. 5d). By targeting p53, miR-22-3p modulates the invasion, metastasis, and proliferation of endometrial epithelial cells.

## Discussion

EMs remains one of the most prevalent gynecological disorders, affecting approximately 6%-10% of reproductive-aged women (Zondervan et al. 2020). EMs is associated with numerous symptoms and effects, such as pelvic pain, dysmenorrhea, painful sex, heavy menstrual bleeding, mental disorders, chronic fatigue, and infertility. Despite being fairly common and having an enormous impact on women’s health, the precise pathophysiology of EMs remains unclear. Owing to its complex pathophysiology, variable symptoms, and inadequate diagnostic methods, diagnosis is often delayed by 4 to 11 years (Taylor et al. 2021). Early diagnosis and treatment are necessary to improve patient outcomes and reduce the recurrence rate, and the development of noninvasive and precise diagnostic biomarkers is urgently needed.

Exosomes contain an abundance of biomolecules that are representative of intercellular communication in the development of cancer (Kalluri et al. 2020). Proteins, lipids, and nucleic acids are abundant in exosomes and could provide novel opportunities to develop less invasive methods for the diagnosis of breast cancer (Zou et al. 2021), colorectal cancer (Ghafouri-Fard et al. 2021), and brain tumors (Ghasempour et al. 2022). Previous studies have reported that various miRNAs, including miR-21, may be applicable as blood-based biomarkers for the noninvasive diagnosis of cholangiocarcinoma (Puik et al. 2017). However, no specific biomarker for endometriosis has been clinically confirmed.

The proliferation, differentiation, and apoptosis of cells are regulated by miRNAs. MiRNAs play essential roles in the development of some diseases and cancer (Smolarz et al. 2022). These miRNAs are highly enriched in exosomes (Li et al. 2022). According to published research, exosomal miR-22-3p from adipose-derived stem cells (ADSCs) phosphorylates the AKT/mTOR axis, promotes Schwann cell (SC) migration and proliferation, and directly suppresses the expression of phosphorylase and tensin homolog deleted on chromosome 10 (PTEN) (Yang et al. 2022). Another published paper reported that by targeting SIRT1 and activating the SIRT1/NF-κB pathway, exosomal miR-22-3p from peritoneal macrophages (pMϕs) improved the invasion, migration, and proliferation of human ectopic endometrial stromal cells (eESCs). It has also been revealed that miR-22-3p might promote the growth, Transwell activity and invasion of endometrial epithelial cells (Zhang et al. 2020). Consequently, miR-22-3p might play important roles in the formation and progression of endometriosis. Our results revealed that the expression of miR-22-3p in exosomes was noticeably greater in the peripheral blood of patients with endometriosis than in that of the healthy controls (*p* < 0.001). According to the ROC curve (AUC = 0.9648), the sensitivity was 93.55%, and the specificity was 84.85%, indicating high predictive diagnostic value and suggesting that exosomal miR-22-3p from peripheral blood may be an essential biomarker for endometriosis diagnosis.

However, the precise mechanisms of endometriosis remain unknown. Our previous research revealed that the stem cell factors Musashi-1 and β-catenin were highly expressed in the shed endometrium and unshed endometrium of the endometriosis group and were positively correlated with the mitochondria, Golgi apparatus, ribosomes, and other cellular organelles of endometrial epithelial cells. These factors were markedly increased and the cells were well developed with vigorous energy metabolism. It was hypothesized that Musashi-1 might inhibit replicative cellular senescence by activating the Wnt/β-catenin signaling pathway, resulting in increased cellular organelle ultrastructure and function of endometriotic endometrial epithelial cells considering their potential for mitosis and proliferation, as well as strong invasive, metastatic, and other malignant functions. All of these functions are closely related to the development of endometriosis (Yu et al. 2018). These findings suggest that the regulation of the cell cycle in endometrial epithelial cells is involved in the development of endometriosis. Apoptosis, a form of programmed cell death, is an essential physiological process, but apoptosis is reduced in endometriosis tissues (Sbracia et al. 2016), and apoptosis-inducing agents may be promising therapeutic agents for endometriosis.

Exosomal miRNAs can regulate angiogenesis and stromal fibroblast activation, especially in the carcinogenesis and development of tumors (Hu et al. 2020, Dohmen et al. 2022). In our study, the results showed that exosomal miR-22-3p could regulate endometrial epithelial cell proliferation, Transwell migration, and invasion by targeting and inhibiting p53. Numerous cellular stressors, such as DNA damage, oncogene activation, hypoxia, replication/translation stress, alterations in cellular metabolism, cell cycle arrest, and apoptosis, have been demonstrated to activate and integrate P53 (Liz 2021). Gene therapies targeting p53 have been developed and are in clinical trials (Garber 2006, Bischoff et al. 1996). Many studies have established that miR-22-3p may be a novel target for treating endometriosis and other diseases (Zhang et al. 2020, Jiao et al. 2024, Yang et al. 2022). Combining standard treatments for endometriosis with exosome-based targeting of miR-22-3p may reduce the suppression of p53 and trigger endometrial epithelial cell death, thus increasing therapeutic efficacy and decreasing the likelihood of recurrence.

While our findings align with previous studies implicating miR-22-3p in endometriosis pathogenesis, several aspects of our study represent novel contributions that differentiate it from prior work. Zhang et al. reported that exosomal miR-22-3p derived from peritoneal macrophages promotes proliferation, migration, and invasion of ectopic endometrial stromal cells through the SIRT1/NF-κB signaling pathway (Zhang et al. 2020). In contrast, our study reveals that circulating exosomal miR-22-3p targets a different downstream pathway-the p53 tumor suppressor pathway-in endometrial epithelial cells, which are the primary cell type responsible for endometriotic lesion formation and maintenance. This cell-type specificity and distinct molecular target are important because epithelial and stromal cells play fundamentally different roles in endometriosis pathogenesis, and therapies targeting epithelial cells may offer complementary clinical benefits. Furthermore, to our knowledge, this is the first study to demonstrate the diagnostic potential of circulating exosomal miR-22-3p as a noninvasive biomarker for endometriosis, providing a potential clinical application that extends beyond mechanistic understanding.

Several limitations of this study should be acknowledged. First, the sample size (*n* = 30 per group), while adequately powered for the primary analyses, remains relatively small, and the ROC analysis (AUC = 0.9648) may be subject to overfitting. Independent validation in larger, multi-center cohorts is warranted. Second, we examined only a single exosomal miRNA (miR-22-3p) and a single target (p53), and the interplay with other signaling pathways requires further investigation. Third, the study was performed primarily using primary endometrial epithelial cells in vitro; in vivo animal models would be necessary to validate the therapeutic relevance of our findings.

## Conclusions

We revealed that exosomal miR-22-3p may promote the growth, migration, and invasion of endometrial epithelial cells and might have a significant impact on the development and progression of endometriosis. Exosomal miR-22-3p may be an essential biomarker for the diagnosis of endometriosis. Combining standard treatments for endometriosis with exosome-based targeting of miR-22-3p may decrease the suppression of p53 and trigger endometrial epithelial cell death. Such an approach could improve treatment efficacy and decrease the likelihood of illness recurrence.

## Data Availability

No datasets were generated or analysed during the current study.
